# Salecan Suppresses Pancreatic Cancer Progression by Promoting Necroptosis via the RIPK1/MLKL Pathway

**DOI:** 10.3390/nu17193090

**Published:** 2025-09-28

**Authors:** Wenya Du, Rong Xu, Pengfei Chen, Jianxia Wen, Luchuanyang Sun, Xianggui Chen

**Affiliations:** School of Food and Bioengineering, Food Microbiology Key Laboratory of Sichuan Province, Chongqing Key Laboratory of Specialty Food Co-Built by Sichuan and Chongqing, Xihua University, Chengdu 610039, China; m18328745397@163.com (W.D.); x1017208563@163.com (R.X.); chenpengfei@xhu.edu.cn (P.C.); wenjx32@163.com (J.W.); slcywelcome@126.com (L.S.)

**Keywords:** pancreatic cancer, Salecan, RNA-seq, necroptosis

## Abstract

**Background/Objectives:** Pancreatic ductal adenocarcinoma (PDAC) is a malignant tumor and leads to high human malignancy and mortality. Because PDAC is highly drug-resistant and current treatments have adverse reactions, exploring novel approaches for PDAC prevention and therapy is urgently needed. **Methods:** Antitumor activities of Salecan were evaluated on multiple human pancreatic adenocarcinoma cells in vitro. Cell viability, colony formation, migration and invasion, flow cytometry, caspase-3 activity, qRT-PCR and Western blotting were monitored. RNA-seq was conducted to clarify the mechanism underlying Salecan’s inhibition of pancreatic cancer cell progression. **Results:** Here we show that Salecan, a naturally occurring polysaccharide of β-glucan, can significantly inhibit pancreatic cancer cell proliferation and exhibit no toxicity in normal cells. We find that Salecan impedes pancreatic cancer cell migration and invasion via the epithelial-to-mesenchymal transition (EMT) pathway. Mechanistically, through RNA sequencing, we reveal that Salecan induces pancreatic cancer cell necroptosis, instead of apoptosis. Moreover, Salecan’s anti-pancreatic cancer bioactivity is attributed to its promotion of the receptor-interacting protein kinase 1 (RIPK1) and mixed lineage kinase-like (MLKL) signaling pathway. **Conclusions:** Salecan can inhibit pancreatic cancer cell proliferation, migration and invasion in vitro and accelerate cell death by inducing the necroptosis via the MLKL/RIPK1 pathway. These findings identify that Salecan may become a potential functional food component for preventing and treating PDAC.

## 1. Introduction

Pancreatic ductal adenocarcinoma (PDAC) is one of the most lethal human malignancies with a poor prognosis, and it is projected to become the second leading cause of cancer-related mortality by 2040 [1]. Due to its late-stage and incurable-stage diagnoses, which make most treatment options ineffective, as well as the fact that current chemotherapy strategies only improve survival by no more than a few months, its 5-year survival rates remain as low as 3–15% [2,3]. Adjuvant chemotherapy drugs for patients with pancreatic cancer include gemcitabine, 5-fluorouracil (5-FU), paclitaxel, etc. Although they are effective in advanced and metastatic patients, significant drug resistance remains a major challenge, along with substantial adverse effects including hematologic toxicity, gastrointestinal reactions, and neurotoxicity [4]. Thus, therapies for improving pancreatic cancer patients’ prognoses lack safe, stable, and low-side-effect approaches [5,6]. Exploring novel avenues against pancreatic cancer is urgently needed. Substantial experimental evidence indicates that natural products may offer protection against cancer in humans [7]. Therefore, investigating natural resources is important for identifying novel candidate drugs to combat pancreatic cancer.

β-glucan, a naturally occurring polysaccharide, has been extensively studied. It contains nutrients and dietary fibers, composed of D-glucose molecules linked by β-1,3 and β-1,4-glycosidic bonds. The macromolecular structure, functions, and applications of β-glucans vary depending on their sources [8]. In recent years, the physiological activities of β-glucans have been elucidated, and they are widely used in the food and pharmaceutical industries due to their nutritional properties [9,10]. β-glucan has been proven to have anti-inflammatory properties and can regulate the immune responses [11]. Moreover, experimental studies and preliminary clinical trials have confirmed the potential efficacy of β-glucan in managing metabolic syndrome, cardiovascular diseases, diabetes, gastrointestinal disorders, cancer, immune-related diseases, and respiratory conditions [12,13,14]. Most studies focus on the preventive and therapeutic capabilities of β-glucan. However, the deep molecular mechanism of β-glucan in diseases remains unclear.

Few studies of β-glucan in PDAC have been conducted. It was found that Antrodia extract contained three effective components, including 1, 3-β-D-glucan, which could inhibit the migration of BxPC-3 pancreatic cancer cells and induce mitochondria-mediated apoptosis [15]. It is suggested that the active ingredient 1, 3-β-D-glucan may have preventive and therapeutic effects on pancreatic cancer.

**Figure 1:**
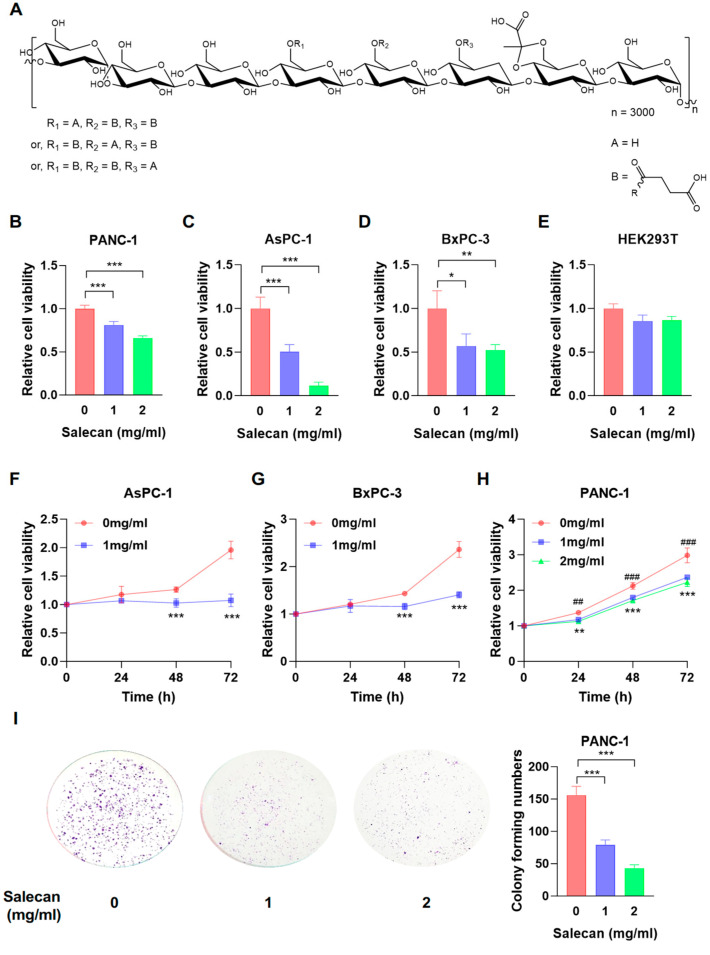
Salecan inhibited the proliferation of pancreatic cancer cells. (**A**) Chemical structure of Salecan. (**B**–**D**) The pancreatic cancer cell lines PANC-1 (**B**), AsPC-1 (**C**), and BxPC-3 (**D**) were treated with the indicated concentrations of Salecan for 48 h; CCK-8 was added, followed by incubation for 2 h, and the absorbance was assessed at 450 nm. (**E**) HEK293T cells were treated with Salecan for 48 h and harvested for CCK-8 assays. (**F**,**G**) AsPC-1 (**F**) and BxPC-3 (**G**) cells were treated with Salecan (0 and 1 mg/mL) for 0, 24, 48, and 72 h and then harvested for CCK-8 assays. (**H**) PANC-1 cells were treated with certain concentrations of Salecan (0, 1, and 2 mg/mL) for 0, 24, 48, and 72 h, then harvested for CCK-8 assays. (**I**) PANC-1 cells were seeded in six-well plates for 24 h and treated with Salecan (0, 1, and 2 mg/mL) for 12 d, followed by a clonogenic assay. All data are presented as the mean ± SEM of three independent experiments. * *p* < 0.05, ** *p* < 0.01, *** *p* < 0.001. ^##^ *p* < 0.01, ^###^ *p* < 0.001.

Salecan is a natural type of β-glucan consisting of nine residues linked by β-(1→3)/α-(1→3) glycosidic bonds with an average molecular weight of 2000 kDa [16] and is one of the novel food ingredients approved in China. Salecan is a water-soluble β-glucan produced by Agrobacterium sp. ZX09 with a unique structure composed of the following repetitive unit: →3)-β-D-Glcp-(1→3)-[β-D-Glcp-(1→3)-β-D-Glcp-(1→3)]3-α-D-Glcp-(1→3)-α-D-Glcp-(1→ (Figure 1A) [17,18]. Salecan has functional food applications, such as in hydrogel dressings [19], food emulsification systems [20], and preservation technologies [21]. In addition, Salecan has a wide range of biological properties, such as improving bacterial infection treatment [22], reducing liver injury [18,23,24,25], alleviating dextran sulfate sodium-induced colitis [26], relieving constipation [27], and decreasing lipid accumulation and metabolism in diet-induced obesity [28]. However, at present, there are no studies reporting on the use of Salecan for cancer treatment, and Salecan’s anti-pancreatic cancer activity has also not been reported. Thus, it is of interest to investigate the potential mechanism of Salecan in PDAC and explore new strategies for PDAC treatment.

The purpose of this study is to investigate the anti-tumor effects of Salecan in PDAC. In this study, Salecan’s anti-pancreatic cancer bioactivity was evaluated, and its mechanism of action through necroptosis was also demonstrated.

## 2. Materials and Methods

### 2.1. β-Glucan

Salecan is a natural glucan from bacteria (*Agrobacterium* sp. ZX09) [29], and it was obtained from Sichuan Synlight Biotech Ltd. (Chengdu, China). A 10 mg/mL stock solution of Salecan was prepared using DEPC-treated water. During the preparation process, heating and stirring were employed to promote dissolution.

### 2.2. Cell Culture

HEK293T, PANC-1, AsPC-1, and BxPC-3 cell lines were purchased from Wuhan Pricella Biotechnology Co., Ltd. (Wuhan, China). HEK293T and PANC-1 cells were cultured in DMEM (Pricella, Wuhan, China), while AsPC-1 and BxPC-3 cells were maintained in RPMI-1640 medium (Pricella, Wuhan, China). The media were supplemented with 10% fetal bovine serum (Pricella, Wuhan, China) and 100 U/mL penicillin–streptomycin solution (Pricella, Wuhan, China). Cells were grown at a temperature of 37 °C in a 5% CO_2_ incubator.

### 2.3. Cell Proliferation

Cell proliferation was assessed using the Cell Counting Kit-8 (CCK-8) assay. In short, cells were seeded in 96-well plates at a density of 5 × 10^3^ cells per well with at least 4 technical replicates overnight. Then, the cells were treated with a certain concentration of Salecan (0, 1, and 2 mg/mL) for 24, 48, and 72 h and incubated with 10 μL of CCK-8 reagent (Biosharp, Hefei, China) at 37 °C for another 2 h; the absorbance at 450 nm was measured using a microplate reader (DeTie, Nanjing, China). The cell viability rates were calculated using the formula provided by the manufacturer of the CCK-8 (Biosharp, Hefei, China). In the rescue experiment, cells were treated with Salecan in the absence or presence of necroptosis inhibitor Necrostatin-1 (Nec-1, MedChemExpress, Monmouth Junction, NJ, USA) for 48 h. And then, the cell viability rates were assessed as mentioned above. The experiments were biologically repeated in triplicate or more.

### 2.4. Colony Formation Assay

This protocol was performed by following previous publications [30,31]. PANC-1 cells (4 × 10^3^ cells/well) were plated in 6-well plates with 3 technical replicates. After incubation overnight, cells were treated with different concentrations (0, 1, and 2 mg/mL) of Salecan for 12 days, with the medium being replaced with a medium containing fresh Salecan every 3 days. Then, the cells were fixed with 4% paraformaldehyde (Biosharp, Hefei, China) and then stained with 0.1% crystal violet (Biosharp, Hefei, China) solution. Cell clusters containing more than 50 cells were regarded as a single clone for counting, and the number of clones was quantified using Image J software (version 1.54 g). The experiments were biologically repeated in triplicate or more.

### 2.5. Wound Healing Assay

PANC-1 cells (2 × 10^5^ cells/well) and BxPC-3 cells (3 × 10^5^ cells/well) were seeded in 6-well plates with 3 technical replicates and allowed to form a confluent monolayer until the density reached 80–90%. The culture medium was then replaced with serum-free medium, and the samples were treated with different concentrations of Salecan. A scratch wound was made using a pipette tip. The cells were then observed and photographed under a microscope (Nikon, Tokyo, Japan) at 0, 24, and 48 h, and data were shown as percentage ratios with the control group. The experiments were biologically repeated in triplicate or more.

### 2.6. Cell Migration and Invasion Assay

For migration and invasion assays, cells were seeded in the upper chamber (invasion: 5 × 10^4^ cells/well; migration: 2 × 10^4^ cells/well) in 200 μL of FBS-free media containing different concentrations of Salecan (0, 1, and 2 mg/mL). The lower chamber contained 700 μL of media supplemented with 10% FBS as a chemoattractant. For invasion assays, the membrane was pre-coated with Matrigel (1:8 dilution in serum-free medium, Beyotime, Shanghai, China), while migration assays used uncoated membranes. After incubation for 24 h, the non-migrating cells in the upper chamber were gently wiped off, and then the transfer chamber was fixed with 4% paraformaldehyde for 30 min, stained with 0.1% crystal violet for 20 min, and washed twice with PBS. Each group was subjected to three independent experiments, observed by an inverted microscope (Nikon, Tokyo, Japan), and quantified with Image J software. The experiments were biologically repeated in triplicate or more.

For the quantification of the results, an inverted microscope was used to take pictures of each well, ensuring that the images covered the entire field of view. The numbers of migrated and invaded cells were quantified using Image J software by counting cells in all fields at 200× magnification. Finally, statistical analysis of the counting results of each replicate experiment was performed. The final results were presented as mean ± SEM.

### 2.7. Flow Cytometry Analysis

Cells were seeded at 3 × 10^5^ cells/well in 6-well plates with 3 technical replicates overnight and treated with Salecan for 48 h. Apoptosis analysis was performed according to the manufacturer’s instructions (eBioscience, San Diego, CA, USA). The cells were harvested, washed with PBS, and resuspended with 1× binding buffer. Afterward, the cell suspension was resuspended in Annexin V-FITC, followed by a 10 min incubation in the dark at room temperature. Subsequently, propidium iodide (PI) was added for a 5 min incubation in the dark. After that, cells were resuspended and immediately subjected to flow cytometry analysis. The data analysis categorized the results as follows: Q2-UL (top left): necrotic cells; Q2-UR (upper right): late apoptotic cells; Q2-LR (bottom right): early apoptosis; and Q2-LL (lower left): cells that did not undergo apoptosis. The apoptosis rate was calculated by summing the percentages from Q2-UR and Q2-LR quadrants. The experiments were biologically repeated in triplicate or more.

### 2.8. Caspase-3 Activity Measurement

Cellular caspase-3 activity was assayed using the Caspase 3 Assay Kit (Beyotime, Shanghai, China) according to the manufacturer’s instructions. Cell lysates treated with Salecan were incubated with Ac-LEVD-pNA for 2 h, and the activity of caspase-3 was determined by spectrophotometry at 405 nm.

### 2.9. RNA-seq Analysis

Cells in the logarithmic growth phase were inoculated in 6-well plates with 3 technical replicates and cultured for 24 h. Subsequently, they were incubated for 48 h with fresh culture media containing different concentrations of Salecan (0 and 2 mg/mL). RNA-seq analysis was performed by TSINGKE Biological Technology Company (Beijing, China). Sequencing libraries were generated using VAHTS Universal V6 RNA-seq Library Prep Kit (Vazyme, Nanjing, China) for Illumina^®^, following the manufacturer’s recommendations. RNA-seq data were deposited into the NCBI Sequence Read Archive (SRA) database and can be accessed through the BioProject accession number RPJNA1263661.

### 2.10. Functional and Pathway Enrichment Analysis

Functional and pathway enrichment analysis of the differential expression genes (DEGs) was conducted using DEseq2 (https://bioconductor.org/packages/release/bioc/html/DESeq2.html, accessed on 21 December 2024; version 1.260). GO and KEGG analyses for the DEGs were conducted. The *p*-values were adjusted using the Benjamini and Hochberg method. A *p*-value less than 0.05 was considered statistically significant.

### 2.11. Quantitative Real-Time PCR

Total RNA of cells was extracted using TRIzol Reagent (MRC, Cincinnati, OH, USA) according to the manufacturer’s protocol. Reverse transcription of RNA to cDNA was conducted using ABScript Neo RT Master Mix for qPCR with gDNA Remover (ABclonal, Wuhan, China). qRT-PCR in triplicate technical replicates was performed using 2× Universal SYBR Green Fast qPCR Mix (ABclonal, Wuhan, China) with specific primers. Data were normalized to the β-actin housekeeping gene level. The relative changes in gene expression were analyzed using the 2^−ΔΔCt^ method. The experiments were biologically and technically repeated in triplicate or more. Primers used are provided in Table S1.

### 2.12. Western Blotting

Cells were seeded in 6-well plates overnight and treated with Salecan (0 and 2 mg/mL) for 48 h. Total proteins of cells were extracted using RIPA lysis buffer (Thermo Fisher Scientific, Waltham, MA, USA) with protease inhibitors (MedChemExpress, NJ, USA) and phosphatase inhibitors (MedChemExpress, NJ, USA), sonicated and centrifuged to remove insoluble material, and determined by BCA Protein Assay Kit (CWbiotech, Beijing, China). Protein samples were separated by SDS-PAGE (10%) and transferred onto PVDF membranes. And then, membranes were incubated with primary antibodies overnight at 4 °C after blocking in 5% milk in TBST for 1 h and then incubated with secondary antibodies for 2 h at room temperature. The following antibodies were used: anti-ZO-1 (1:5000, Proteintech, Wuhan, China), anti-MMP-2 (1:1000, ABclonal, Wuhan, China), anti-Snail (1:500, ABclonal, Wuhan, China), anti-RIPK1 (1:1000, ABclonal, Wuhan, China), anti-MLKL (1:1000, ABclonal, Wuhan, China), anti-p-RIPK1 (1:1000, Nature Biosciences, Hangzhou, China), anti-p-MLKL (1:1000, Nature Biosciences, Hangzhou, China), β-actin (1:10,000, Proteintech, Wuhan, China), and GAPDH (1:50,000, Proteintech, Wuhan, China). Western blotting bands were quantified by Image J software. The experiments were biologically and technically repeated in triplicate or more.

### 2.13. Statistical Analysis

The data are presented as mean ± SEM from at least three independent experiments. Analysis of the data was performed using GraphPad Prism version 10.0 (GraphPad Software Inc., San Diego, CA, USA). The unpaired two-tailed Student’s *t* test was used to determine the statistical difference between the two groups, while statistical significance among multiple groups was determined using one-way or two-way analysis of variance. *p*-value < 0.05 was significant.

## 3. Results

### 3.1. Salecan Inhibits Pancreatic Cancer Cell Proliferation

Cell proliferation capacity is a critical indicator used to assess the anti-tumor ability of drugs and drug-active substances [32]. Cell proliferation assays were conducted to evaluate the effects of Salecan on pancreatic cancer cells at the concentrations of 1 and 2 mg/mL, which were determined based on another study [33]. Results revealed that Salecan effectively suppressed the activity of PANC-1, AsPC-1, and BxPC-3 after treatment within 48 h (Figure 1B–D), while Salecan exhibited no significant cytotoxicity on normal human embryonic kidney 293T cells (HEK293T) (Figure 1E). Moreover, AsPC-1 and BxPC-3 cells were incubated with a concentration of 1 mg/mL of Salecan for 24, 48, and 72 h to verify cell proliferation capabilities, and the results showed that the inhibitory ability of Salecan on cells acted in a time-dependent manner (Figure 1F,G). In addition, in PANC-1 cells, Salecan at concentrations of 1 and 2 mg/mL exhibited an increasing inhibition of viability, showing time- and concentration-dependent patterns (Figure 1H). These results suggest that Salecan inhibits cell proliferation in multiple pancreatic cancer cell lines.

A colony formation assay is another method of determining cell survival and the proliferation ability of individual cells [34]. Therefore, the colony formation assay was performed to assess the effect of Salecan on the proliferation ability of PDAC cells. PANC-1 cells were treated with Salecan at concentrations of 1 and 2 mg/mL, and then the clone numbers were calculated after 12 days. The results revealed that Salecan inhibited the colony formation of PANC-1 cells (Figure 1I) in a concentration-dependent manner. All these results confirmed that Salecan can suppress pancreatic cancer cell proliferation.

### 3.2. Salecan Attenuates Pancreatic Cancer Cell Migration and Invasion in a Concentration-Dependent Manner

**Figure 2:**
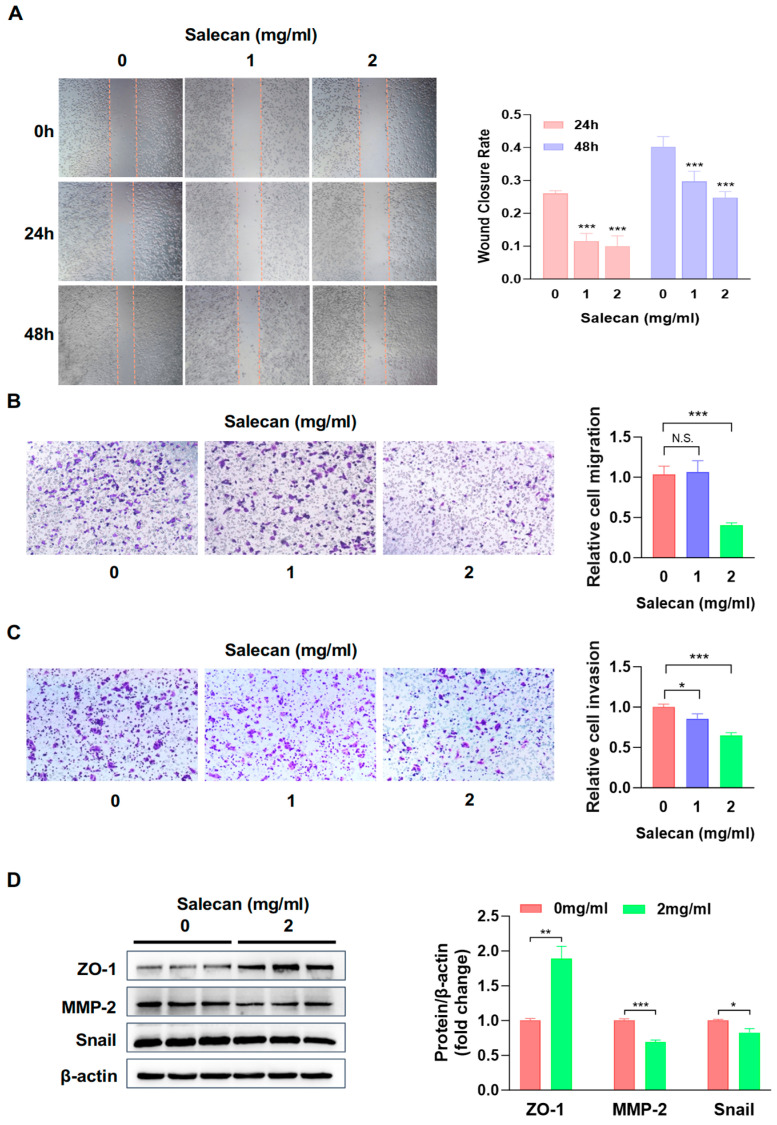
Salecan suppressed pancreatic cancer cell migration and invasion. (**A**) Wound healing analysis of PANC-1 cell migration treated with Salecan at indicated concentrations for 0, 24, and 48 h. (**B**,**C**) Transwell assay of PANC-1 cell migration (**B**) and invasion (**C**) treated with Salecan with indicated concentrations for 24 h. (**D**) The protein expression levels of ZO-1, Snail, and MMP-2 were determined by Western blotting after treatment with Salecan for 48 h. Quantitative results were normalized to β-actin. All data are presented as the mean ± SEM of three independent experiments. * *p* < 0.05, ** *p* < 0.01, *** *p* < 0.001.

Pancreatic cancer cells are highly aggressive and migratory, and these traits lead to early metastasis. They also reduce the effectiveness of treatments; therefore, developing novel therapeutic strategies to combat high invasiveness and metastasis is urgently needed [35]. Next, we attempted to elucidate Salecan’s ability to suppress pancreatic cancer cell metastasis. Firstly, wound-healing assays were used to investigate the effect of Salecan on PANC-1 cell migration. We found that compared with the control group, samples treated with Salecan could decrease the migration ability of PANC-1 cells in a dose-dependent manner (Figure 2A); the same effect was observed for BxPC-3 cell migration (Figure S1A). Transwell assays were employed to evaluate Salecan’s effects on the migration and invasion capabilities of PANC-1 cells, with the results indicating that it had a reducing effect on PANC-1 cells’ migration and invasion abilities in a dose-dependent manner (Figure 2B,C). These results revealed that Salecan could significantly restrain pancreatic cancer cell migration and invasion.

The epithelial-to-mesenchymal transition (EMT) is a complex process in cellular transformation and plays a pivotal role in tumor progression and self-renewal, as well as in cancer metastasis [36]. There are several molecular markers involved in the EMT process, such as zonula occludens-1 (ZO-1). ZO-1 is a tight junction ligand protein, and its expression decreases in tumor cells [37]. Matrix metalloprotease 2 (MMP-2) is involved in invasive blood vessel growth and promotes metastasis [38]. Snail is a transcriptional regulator of E-cadherin and induces the EMT [36]. We used Western blotting analysis to verify the protein expression of ZO-1, MMP-2, and Snail, and the results showed ZO-1 protein levels were significantly elevated; however, MMP-2 and Snail levels were remarkably decreased after treatment with Salecan in PANC-1 cells (Figure 2D) and BxPC-3 cells (Figure S1B).

Taken together, these results demonstrated that Salecan suppressed pancreatic cancer cell migration and invasion through the EMT-associated signaling pathway.

### 3.3. RNA-seq Analysis Reveals That Salecan Affects the Necroptosis Pathway, Not Apoptosis, in Pancreatic Cancer Cells

**Figure 3:**
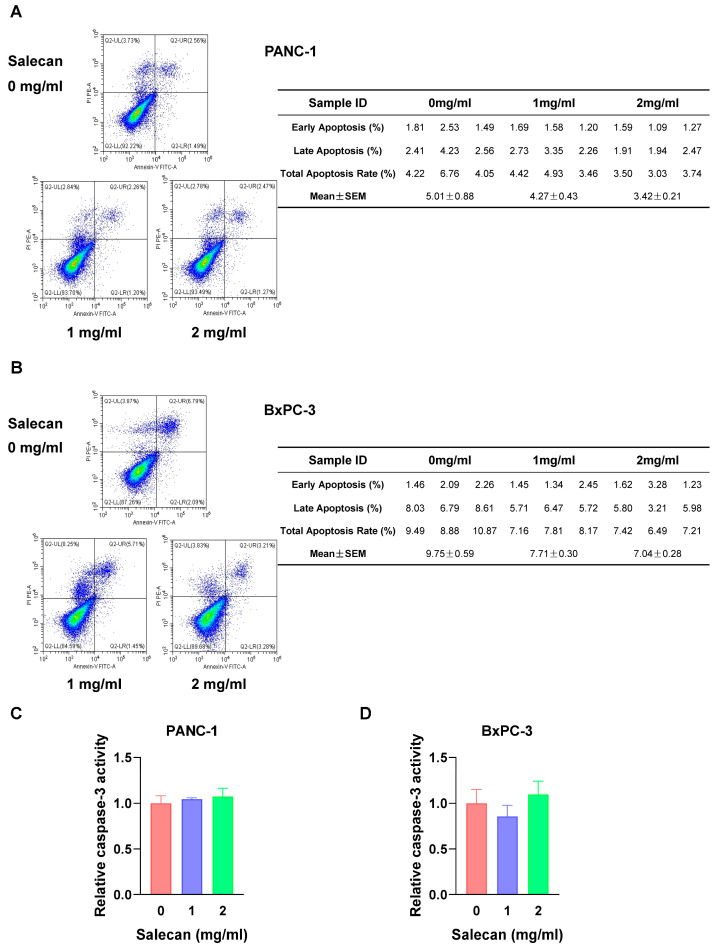
Apoptosis of pancreatic cancer cells was not affected by Salecan. (**A**,**B**) Apoptosis was evaluated and quantified by flow cytometry in PANC-1 cells (**A**) and BxPC-3 cells (**B**) treated with different concentrations of Salecan for 48 h. The table comprehensively lists the calculated apoptotic rates with Salecan. (**C**,**D**) Caspase-3 activity was determined in PANC-1 cells (**C**) and BxPC-3 cells (**D**) treated with different concentrations of Salecan for 48 h.

Apoptosis, as one of the cell death mechanisms, is critical in cancer development. Most drugs inhibit tumors by promoting tumor cell apoptosis [39,40]. Flow cytometry was employed to determine whether Salecan could promote apoptosis in PANC-1 cells and BxPC-3 cells. Annexin-V/PI staining can be used to quantify apoptotic cell numbers. It is interesting that there was no significant effect of apoptosis on pancreatic cancer cells treated with Salecan in PANC-1 cells (Figure 3A) and BxPC-3 cells (Figure 3B). To further verify these results, the activation of caspase-3 was determined, and the results showed that Salecan did not increase the activity of caspase-3 in both cell lines (Figure 3C,D). Thus, to understand Salecan’s influence on the proliferation and metastasis of pancreatic cancer cells, we determined its effect via RNA sequencing (RNA-seq) and comprehensively analyzed transcriptome datasets, including the Kyoto Encyclopedia of Genes and Genomes (KEGG) and Gene Ontology (GO) datasets.

**Figure 4:**
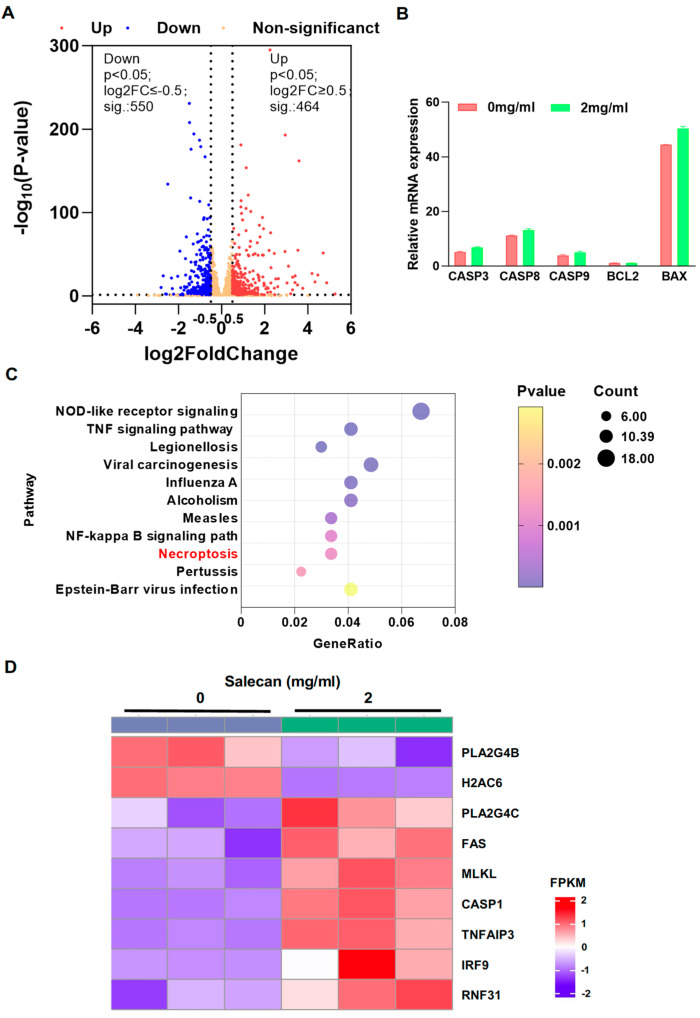
Salecan ameliorated the proliferation of pancreatic cancer cells by targeting necroptosis. (**A**) The volcano plot shows the total expression of mRNAs in PANC-1 cells. Orange indicates the mRNAs with non-significant differences; red and blue indicate those with significant differences. (**B**) Analyzed mRNA expression levels of several crucial apoptosis-related factors (CASP3, CASP8, CASP9, BCL2, and BAX) between the Ctrl and Salecan-treated group in PANC-1 cells using transcriptome data, with FPKM as the normalization method for gene expression levels. (**C**) Top KEGG pathways are shown for Salecan versus Ctrl derived from (**A**). (**D**) Heat maps demonstrating necroptosis gene expression in Salecan versus Ctrl derived from (**A**).

Hence, to clarify the mechanism underlying Salecan’s inhibition of pancreatic cancer cell progression, transcriptome analysis was performed on PANC-1 cells treated with Salecan, and these cells were compared to the control groups. The volcano map showed that 464 and 550 genes were upregulated and downregulated (|Log2FC| ≥ 0.5, *p*-value < 0.05), respectively, in Salecan-treated cells compared with the control group (Figure 4A). Based on the previous results, there was no difference in apoptosis between the Salecan-treated and control groups, and after searching the transcriptome datasets for apoptosis-related factors, including the caspase and Bcl families, we further demonstrated that there were no significant differences in the molecular markers of apoptosis (Figure 4B), which was consistent with the above results. Furthermore, pathway enrichment analysis showed that the necroptosis signaling pathway was highly enriched (Figure 4C), and the heat map revealed that MLKL, an important factor in necroptosis, had significantly increased in pancreatic cancer cells treated with Salecan (Figure 4D). These data collectively indicated the pivotal role of Salecan in inducing necroptosis, not apoptosis, and impeding cell proliferation.

### 3.4. Salecan Impairs Pancreatic Cancer Cell Proliferation and Metastasis via the MLKL/RIPK1 Signaling Pathway

**Figure 5:**
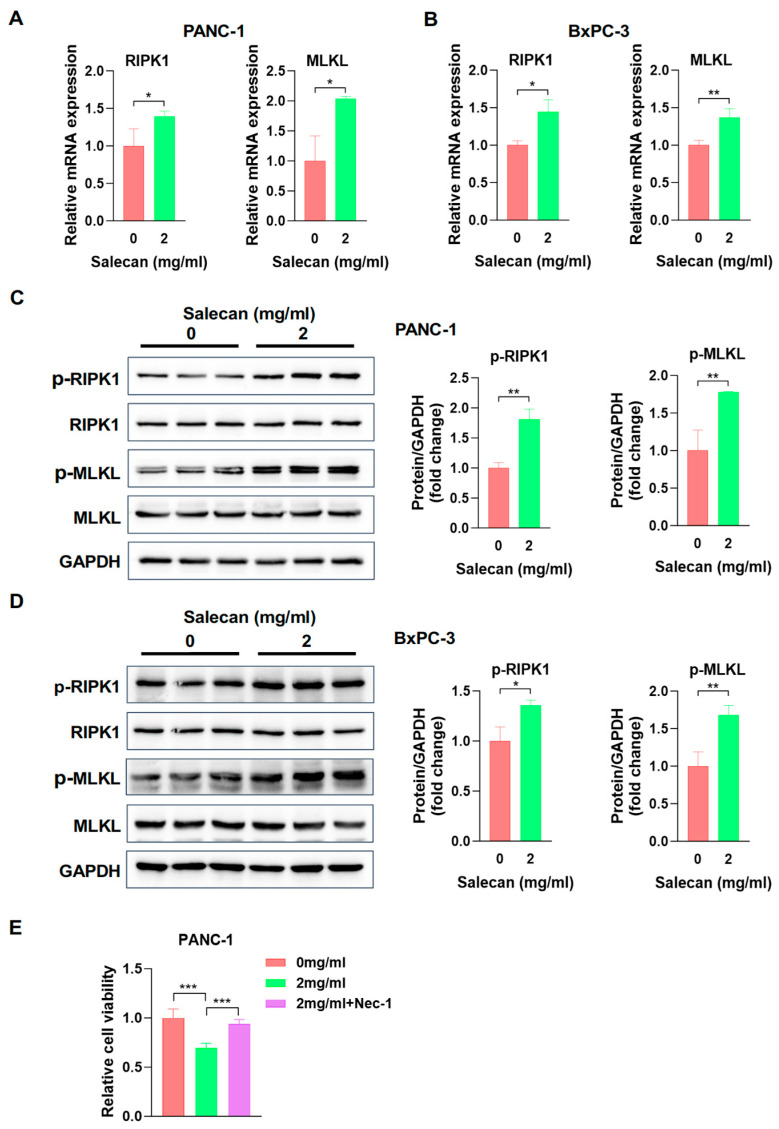
Salecan promoted the RIPK1/MLKL signaling pathway. (**A**,**B**) qRT-PCR analysis of RIPK1 and MLKL mRNA expression in PANC-1 (**A**) and BxPC-3 (**B**) cells treated with Salecan for 48 h. (**C**,**D**) The cell necroptosis signaling pathway molecules were detected by Western blotting with Salecan after 48 h in PANC-1 (**C**) cells and BxPC-3 cells (**D**). Quantitative results were normalized to GAPDH. (**E**). PANC-1 cells were treated with Salecan and 50 μM Nec-1 for 48 h and then harvested for CCK-8 assays. All data are presented as the mean ± SEM of three independent experiments. * *p* < 0.05, ** *p* < 0.01, *** *p* < 0.001.

**Figure 6:**
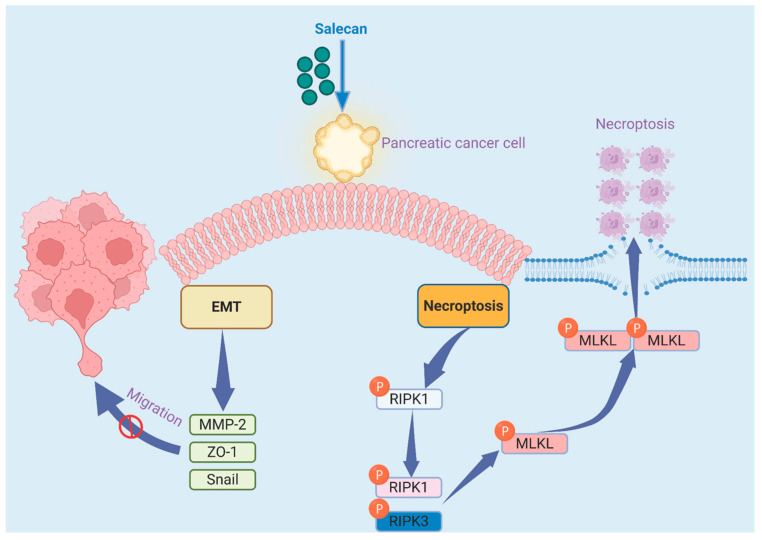
Salecan suppressed cell proliferation and metastasis via the RIPK1/MLKL pathway.

Receptor-interacting protein kinase 1 (RIPK1) and mixed lineage kinase-like (MLKL) are two core genes related to necroptosis. To further explore the influence of Salecan on necroptosis, we evaluated the mRNA expression of RIPK1 and MLKL in PANC-1 cells, and both were significantly upregulated (Figure 5A), which was consistent with the transcriptome data from the RNA-seq analysis. The same result was also obtained in BxPC-3 cells (Figure 5B). In addition, the most important step in necroptosis is RIPK1 and RIPK3 phosphorylation, which activates MLKL; then, the phosphorylated MLKL translocates to the plasma membrane, causing cell membrane rupture, swelling, and the loss of cell and organelle integrity [41]. Therefore, we further verified and compared the protein levels of p-RIPK1 and p-MLKL in the Salecan-treated group compared to the control group, and both were increased in the Salecan-treated group in PANC-1 and BxPC-3 cells according to the Western blotting (Figure 5C,D). To further confirm that the inhibitory effect of Salecan is derived from necroptosis, we used necroptosis inhibitor Necrostatin-1 (Nec-1) to carry out cell proliferation experiments in PANC-1 cells [42]. Cell proliferation assays confirmed that the decreases in cell viability after Salecan treatment were significantly restored by the inhibition of necroptosis by Nec-1 (Figure 5E). These results strongly suggested that Salecan activated the MLKL/RIPK1 signaling pathway, impairing pancreatic cancer cell proliferation and metastasis (Figure 6).

## 4. Discussion

In this study, we showed that Salecan could significantly inhibit pancreatic cancer cell proliferation by promoting cell necroptosis, thereby decreasing the occurrence and progression of pancreatic cancer. In the clinic, gemcitabine or FOLFIRINOX is recognized as the preferred first-line therapy to prolong patients’ survival [43,44]. However, chemical drugs are often associated with adverse effects or toxicity. In addition, drug resistance in PDAC is also a severe clinical problem, as a lot of these patients relapse within a few months after receiving the standard-of-care chemotherapy [43,45]. Fortunately, as a dietary fiber, Salecan has no toxicity and is beneficial for human health [46]. In addition, Salecan showed good anti-tumor activity and decreased the progression of multiple pancreatic cancer cell lines, like human pancreatic ductal adenocarcinoma cell lines (PANC-1 and BxPC-3) and the human pancreatic adenocarcinoma cell line AsPC-1, in our research. Moreover, we found that Salecan increases pancreatic cancer cell necroptosis, thereby inhibiting cell proliferation. Although Salecan has various biological effects, such as inducing liver injury [18,24] and lung injury [47] and anti-fatigue effects [17], no one has reported Salecan’s bioactivity against cancer, or against pancreatic cancer cell proliferation in particular. Our results indicate that Salecan may be a promising functional food for the management of pancreatic cancer. However, this study evaluated the anti-cancer potential of Salecan against pancreatic cancer in vitro. It should be noted that in vivo experiments have not been conducted yet. In the future, we will conduct more in vivo experiments and clinical studies to help develop Salecan into a functional food for preventing pancreatic cancer. Furthermore, our following research will involve a comparative analysis of the pharmacological activities of Salecan and other clinical drugs, with a particular emphasis on exploring whether combination therapy could potentially enhance clinical outcomes in pancreatic cancer management. As there are no reports on the application of Salecan in cancer treatment, it is of great significance to further enhance the research on Salecan in pancreatic cancer and assess the clinical efficacy of combination therapies for this disease. Beyond that, exploring the effects of Salecan in other types of cancer and elucidating its underlying mechanism can contribute to unlocking the potential of Salecan in the treatment of cancer. Undoubtedly, these efforts will play a crucial part in future cancer treatment.

Pancreatic adenocarcinoma is the most lethal type of solid tumor; in addition, migration and invasion are characteristic of PDAC occurrence and development [48,49]. During our study, we verified that Salecan attenuates pancreatic cancer cell migration and invasion. Additionally, Salecan could suppress the migration and invasion abilities of PANC-1 cells in a dose-dependent manner. Numerous studies have suggested that the EMT is crucial for PDAC invasion and metastasis [49,50,51]. The following experiments proved that in the Salecan-treated group, the expression levels of MMP-2 and Snail, members of EMT-associated signaling pathways, are decreased, and the expression of ZO-1 is increased. Therefore, Salecan could attenuate pancreatic cancer cell migration and invasion by inhibiting EMT-associated proteins; these EMT inhibitions have a significant impact on the migration and invasion of pancreatic cancer. Controlling the process of EMT with Salecan is also one of the mechanisms of inhibiting pancreatic cancer.

In recent years, researchers have focused on identifying natural active components to combat pancreatic cancer. Some studies were conducted to explore the function of polysaccharides in pancreatic cancer, such as those isolated from Strongylocentrotus nudus eggs [52], Lycium ruthenicum Murr [53], Inonotus obliquus [54], etc. However, most studies on the mechanisms of inhibiting pancreatic cancer found that apoptosis [54,55,56] and autophagy [57] were promoted in pancreatic cancer cells, with no confirmed evidence regarding an impact on necroptosis.

In general, apoptosis is a hallmark of cancer [58], and as research continues, multiple types of regulated cell death have been identified, including necroptosis, pyroptosis, ferroptosis, and alkaliptosis [59]. Necroptosis is a regulated type of programmed cell death. In recent years, studies have confirmed that necroptosis is closely related to PDAC, being involved in tumor growth [60], pancreatic cancer liver metastasis [61,62], and the tumor microenvironment [63]. In this study, apoptosis exhibited no difference in the pancreatic cell lines between the Salecan-treated and control groups. However, previous data confirmed that cell proliferation was inhibited with Salecan. To verify the mechanism of Salecan’s protective effects, changes in global gene expression in PDAC were determined via RNA-seq, and there were significant differences in the regulatory pathway of necroptosis. Necroptosis is executed by RIPKs and MLKL [64]. Phosphorylated RIPKs and MLKL are crucial biomarkers of the necroptosis process [60,65]. In this study, we verified that Salecan could increase the mRNA levels of MLKL and RIPK1, consistent with RNA sequencing results. Then, we found that Salecan could increase RIPK1/MLKL phosphorylation in PANC-1 cells and BxPC-3 cells, suggesting that Salecan can promote necroptosis of pancreatic cancer cells to exert its anti-pancreatic cancer activity. Furthermore, necroptosis inhibitor experiments also confirmed this conclusion. Therefore, Salecan’s inhibition effect on pancreatic cancer cells stems from its promotion of necroptosis, not apoptosis, which is different from the research mechanisms of other polysaccharides in pancreatic cancer. Furthermore, necroptosis inhibits cell metastasis [66], as confirmed by Salecan’s ability to promote necroptosis, thereby alleviating the EMT in pancreatic cancer.

We conducted transcriptome sequencing to investigate the mechanisms used by Salecan to reduce proliferation and metastasis in pancreatic cancer cells. A comprehensive exploration of Salecan’s mechanisms is crucial for understanding the role of functional foods in pancreatic cancer pathogenesis. In subsequent studies, we will continue to delve into the underlying mechanisms to advance the development of drugs and functional foods for pancreatic cancer intervention.

## 5. Conclusions

In summary, this work demonstrates that Salecan can inhibit pancreatic cancer cell proliferation in vitro. Further, Salecan could suppress pancreatic cancer cell migration and invasion, and it could accelerate cell death by inducing the necroptosis signaling pathway. Moreover, Salecan can significantly increase the expression levels of phosphorylated MLKL and RIPK1, which affect pancreatic cell necroptosis. In conclusion, these findings highlight that Salecan may be developed as a potential functional food component to fight pancreatic cancer.

## Data Availability

The original contributions presented in this study are included in the article/Supplementary Materials. Further inquiries can be directed to the corresponding author.

## Supplementary Materials

The following supporting information can be downloaded at https://www.mdpi.com/article/10.3390/nu17193090/s1, Figure S1: Salecan inhibited the migration and EMT of the BxPC-3 cell. Table S1. Primers for qRT-PCR analysis.
