# Supplementary material for: Salecan Suppresses Pancreatic Cancer Progression by Promoting Necroptosis via the RIPK1/MLKL Pathway
Source: Nutrients. 2025 Sep 28;17(19):3090. doi: 10.3390/nu17193090 (PMC12525719; doi:10.3390/nu17193090)
Supplement: Supplementary file 1 [file nutrients-17-03090-s001.zip › nutrients-3842460-supplementary.pdf]

Figure S1

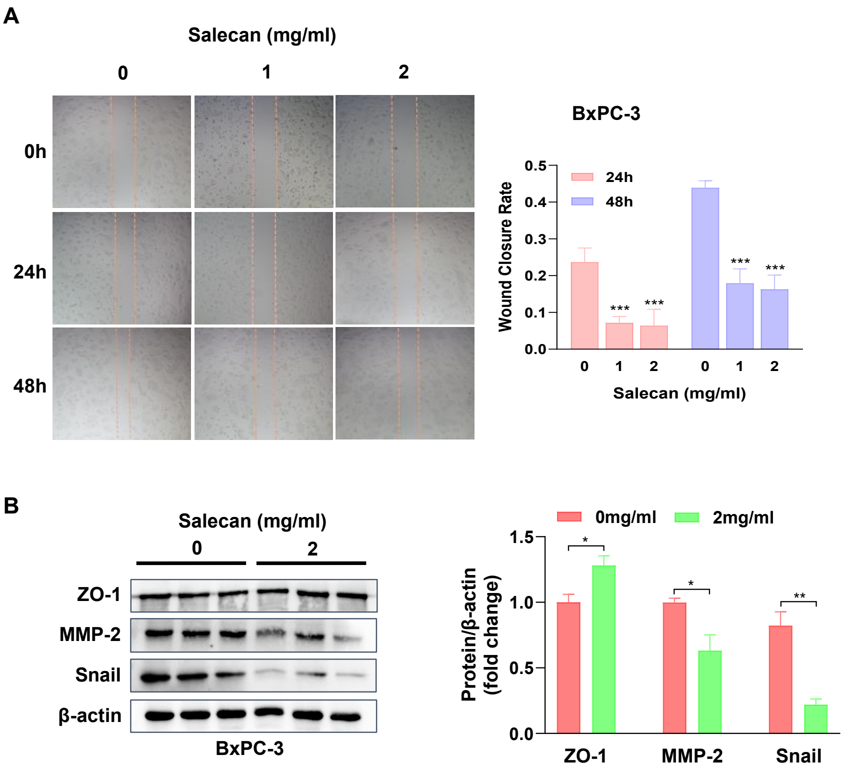

Figure S1: Salecan inhibited the migration and EMT of the BxPC-3 cell.

A. Wound healing analysis of BxPC-3 cells migration treated with Salecan at indicated concentrations for 0, 24 and 48 h . B. In BxPC-3 cells, the protein expression levels of ZO-1, Snail and MMP-2 were determined by Western blotting treated with Salecan after 48 h. Quantitative result was normalized to β-actin. All data were presented as the mean ± SEM of three independent experiments. * $p < 0.05$, ** $p < 0.01$, *** $p < 0.001$.

Table S1. Primers for qRT-PCR analysis

hβ-actin	F	GATTCCTATGTGGGCGACGA
	R	AGGTCTCAAACATGATCTGGGT
hRIPK1	F	GGGAAGGTGTCTCTGTGTTTC
	R	CCTCGTTGTGCTCAATGCAG
hMLKL	F	GTGGGAAAGAAGGTGGAAGAG
	R	GCCAAGGGTGATAATATGCTTC
